# Individual- and Community-Level Predictors of Birth Preparedness and Complication Readiness: Multilevel Evidence from Southern Ethiopia

**DOI:** 10.3390/epidemiologia7010013

**Published:** 2026-01-14

**Authors:** Amanuel Yoseph, Lakew Mussie, Mehretu Belayineh, Francisco Guillen-Grima, Ines Aguinaga-Ontoso

**Affiliations:** 1 Department of Epidemiology and Biostatistics, Schulich School of Medicine & Dentistry, Western University, London, ON N6G 2C6, Canada; 2 School of Public Health, College of Medicine and Health Sciences, Hawassa University, Hawassa P.O. Box 5, Ethiopia; mehretu@hu.edu.et; 3 School of Public Health, University of Saskatchewan, Saskatoon, SK S7N 5A2, Canada; 4 Adare General Hospital, Hawassa City Administration, Hawassa P.O. Box 5, Ethiopia; lakew365@gmail.com; 5 Department of Preventive Medicine, Clinica Universidad de Navarra, 31008 Pamplona, Spain; frguillen@unav.es; 6 Department of Health Sciences, Public University of Navarra, 31008 Pamplona, Spain; 7 Group of Clinical Epidemiology, Area of Epidemiology and Public Health, Healthcare Research Institute of Navarre (IdiSNA), 31008 Pamplona, Spain; 8 CIBER in Epidemiology and Public Health (CIBERESP), Institute of Health Carlos III, 46980 Madrid, Spain

**Keywords:** birth preparedness, complication readiness, multilevel analysis, maternal health, Ethiopia

## Abstract

**Background/Objectives**: Birth preparedness and complication readiness (BPCR) is a cornerstone of maternal health strategies designed to minimize the “three delays” in seeking, reaching, and receiving skilled care. In Ethiopia, uptake of BPCR remains insufficient, and little evidence exists on how individual- and community-level factors interact to shape preparedness. This study assessed the determinants of BPCR among women of reproductive age in Hawela Lida district, Sidama Region. **Methods**: A community-based cross-sectional study was conducted among 3540 women using a multistage sampling technique. Data were analyzed with multilevel mixed-effect negative binomial regression to account for clustering at the community level. Adjusted prevalence ratios (APRs) with 95% confidence intervals (CIs) were reported to identify determinants of BPCR. Model fitness was assessed using Akaike’s Information Criterion (AIC), the Bayesian Information Criterion (BIC), and log-likelihood statistics. **Results**: At the individual level, women employed in government positions had over three times higher expected BPCR scores compared with farmers (AIRR = 3.11; 95% CI: 1.89–5.77). Women with planned pregnancies demonstrated higher BPCR preparedness (AIRR = 1.66; 95% CI: 1.15–3.22), as did those who participated in model family training (AIRR = 2.53; 95% CI: 1.76–4.99) and women exercising decision-making autonomy (AIRR = 2.34; 95% CI: 1.97–5.93). At the community level, residing in urban areas (AIRR = 2.78; 95% CI: 1.81–4.77) and in communities with higher women’s literacy (AIRR = 4.92; 95% CI: 2.32–8.48) was associated with higher expected BPCR scores. These findings indicate that both personal empowerment and supportive community contexts play pivotal roles in enhancing maternal birth preparedness and readiness for potential complications. Random-effects analysis showed that 19.4% of the variance in BPCR was attributable to kebele-level clustering (ICC = 0.194). The final multilevel model demonstrated superior fit (AIC = 2915.15, BIC = 3003.33, log-likelihood = −1402.44). **Conclusions**: Both individual- and community-level factors strongly influence BPCR practice in southern Ethiopia. Interventions should prioritize women’s empowerment and pregnancy planning, scale-up of model family training, and address structural barriers such as rural access and community literacy gaps. Targeted, multilevel strategies are essential to accelerate progress toward improving maternal preparedness and reducing maternal morbidity and mortality.

## 1. Introduction

Maternal and neonatal mortality continues to pose a major public health challenge in sub-Saharan Africa, with the region accounting for nearly two-thirds of global maternal deaths. Recent estimates indicate that the maternal mortality ratio (MMR) in sub-Saharan Africa exceeds 500 deaths per 100,000 live births, substantially higher than the global average of approximately 210 per 100,000 live births [1,2]. Ethiopia bears a disproportionate share of this burden, with maternal mortality remaining persistently high despite sustained investments in maternal health services. Limited access to skilled care, delayed recognition of obstetric complications, and inadequate birth preparedness continue to contribute to preventable maternal and neonatal deaths in the country [3,4,5].

Birth Preparedness and Complication Readiness (BPCR) is a proactive strategy designed to enhance timely access to skilled care and minimize delays in responding to obstetric emergencies [6,7,8]. Core components of BPCR include identifying a skilled birth attendant, arranging transportation to health facilities, saving funds for delivery and emergencies, and recognizing key obstetric danger signs [9,10,11]. Evidence from national surveys and community-based studies in Ethiopia indicates that BPCR practice remains suboptimal, particularly in rural and underserved settings, where a substantial proportion of women fail to complete even basic preparedness actions [12,13,14]. These gaps underscore the continued vulnerability of women to avoidable complications during pregnancy and childbirth.

Individual-level determinants of BPCR have been widely documented, including maternal education, age, parity, employment status, antenatal care attendance, and autonomy in household decision-making [15,16,17,18]. Educated and employed women are consistently more likely to engage in BPCR, as they tend to have better access to health information, financial resources, and decision-making power [16,17]. Similarly, regular antenatal care visits provide critical opportunities for counseling, early detection of complications, and promotion of preparedness behaviors, thereby increasing BPCR adherence [18,19,20]. However, much of the existing literature has focused predominantly on individual-level predictors, often overlooking the broader social and contextual environments in which women live.

Community-level factors—including literacy levels, poverty, exposure to mass media, and proximity to health facilities—play a crucial role in shaping BPCR behaviors [18,20,21]. Women residing in communities with higher literacy and socio-economic status are more likely to practice BPCR, even after accounting for individual characteristics [14,15]. In contrast, communities characterized by limited infrastructure, low literacy, and economic hardship often exhibit lower engagement in BPCR activities [11,12,13,14,15,16,17,18]. Understanding the interaction between individual- and community-level determinants is therefore essential for designing effective, context-sensitive maternal health interventions. Nevertheless, relatively few studies in Ethiopia have examined these multilevel influences simultaneously [18,19,22].

Methodologically, many previous studies have relied on conventional regression approaches that do not adequately account for the hierarchical structure of community-based data, potentially leading to biased estimates and underestimated contextual effects [8,18,23]. Multilevel modeling offers a robust analytical framework to disentangle individual- and community-level influences while accounting for clustering within communities [8,18,23,24]. Such approaches are increasingly recognized as critical for informing targeted interventions and evidence-based health policy decisions.

The Northern Zone of the Sidama Region represents a particularly relevant context for examining BPCR. The area is characterized by marked socio-economic diversity, predominantly rural settlement patterns, variable community literacy levels, and persistent barriers to accessing skilled maternal health services [8,22,25]. Available evidence suggests that BPCR practices in this region remain uneven and insufficient, yet empirical data specific to the Northern Zone are scarce compared with other parts of Ethiopia. This lack of localized evidence limits the ability of policymakers and program planners to design interventions that reflect the region’s unique contextual realities.

Therefore, this study aims to assess the individual- and community-level determinants of BPCR among women of reproductive age in the Northern Zone of Sidama Region, Ethiopia. Using a multistage sampling strategy and multilevel mixed-effects negative binomial regression modeling, the study seeks to generate comprehensive evidence on the factors influencing BPCR practices. The findings are expected to inform context-specific interventions and policy actions to improve maternal preparedness and contribute to reductions in maternal and neonatal morbidity and mortality in the region.

## 2. Materials and Methods

### 2.1. Study Setting

The research was undertaken in Hawela Lida District, one of 30 districts in Ethiopia’s Sidama Region, located approximately 289 km south of Addis Ababa. The district comprises 11 rural and two urban kebeles (the most minor administrative units). In 2024, its estimated population was 131,848 across 24,281 households, with women of reproductive age (WRA) representing 24.3%. Agriculture is the principal economic activity, with major crops including enset (false banana, a staple food), maize, coffee, khat (a locally chewed stimulant plant), barley, haricot beans, sweet potatoes, and indigenous cabbage. Health infrastructure includes 20 government health posts, four health centres, five private medium clinics, two NGO-run facilities, and six private drug stores, staffed by 482 health professionals. Health posts, operated by Health Extension Workers (HEWs), provide Maternal, Newborn and Child Health services such as health education, nutrition screening, antenatal and postnatal care, and family planning. Access to maternity services varies considerably: women residing in urban areas typically have shorter travel times to health facilities, whereas rural women often face long distances, limited transport, and financial constraints. Consequently, home births remain common among women with lower educational attainment and poorer socioeconomic status, reflecting structural and social barriers to facility-based delivery. Overall service coverage is estimated at 70%.

### 2.2. Study Design and Period

A community-based cross-sectional survey was conducted between 1 February and 30 March 2025, in the Hawela Lida district of the Sidama Region, Ethiopia. This period was selected deliberately because it represents a time of routine maternal health service delivery, with no major public health campaigns, policy changes, or seasonal disruptions affecting maternal and child health services. Conducting the study during this stable period helped ensure that the observed BPCR practices reflect usual behaviors and program conditions rather than temporary or atypical influences. The study follows the STROBE guidelines (Supplementary File S1).

### 2.3. Source and Study Populations

The source population comprised all WRA residing in the district. To establish a comprehensive sampling frame, a census of women who had delivered a live infant within the preceding five years was conducted in the selected kebeles. From this frame, the study population for analysis included women whose most recent pregnancy resulted in a live birth, ensuring consistency in reporting of BPCR practices and minimizing recall bias.

### 2.4. Eligibility Criteria

Women were eligible if they had resided in the district for at least six months and experienced a live birth within the preceding five years. Women who were critically ill or had mental health conditions that prevented effective communication were excluded. BPCR was assessed specifically for each woman’s most recent pregnancy.

### 2.5. Sample Size Determination

The sample size was estimated using OpenEpi v3.0 software. Assuming a BPCR prevalence of 33% from a prior study, a 95% confidence level, 5% margin of error, a design effect of 2, and a 10% allowance for non-response, the minimum required sample was 748. To ensure adequate power for identifying determinants of BPCR practice, we additionally considered evidence from a study conducted in Gondar, which suggested a substantially larger sample size. Accordingly, a final sample of 3540 women was retained for this study.

### 2.6. Sampling Procedure

A multistage sampling technique was employed. First, 10 of the district’s 13 kebeles were randomly selected. Within these kebeles, a census identified all women who had delivered a live infant in the past five years, forming the sampling frame. Households were then systematically selected proportional to kebele size. In households with multiple eligible women, one mother was randomly chosen. Households were considered non-respondent after three unsuccessful visits. Although the sampling frame included deliveries in the past five years, BPCR outcomes were measured only for the most recent pregnancy to ensure accurate recall and reliable reporting. Exact time since the last birth was not recorded; however, all questions referred to the most recent pregnancy, allowing standardized measurement across participants. Of the 3540 eligible women identified through the household census, 3526 participated in the survey, yielding a response rate of 99.6%. Non-response was primarily due to temporary absence from the household after three repeated visits or inability to complete the interview at the time of data collection.

### 2.7. Study Variables

The primary outcome variable was BPCR, operationalized as a count measure based on women’s self-reported practices related to their most recent pregnancy. BPCR was assessed using five components: identification of a nearby health facility for childbirth, selection and prior communication with a skilled birth attendant, saving money or material resources for delivery and related expenses, arranging transportation in advance for delivery or obstetric emergencies, and identifying a potential compatible blood donor if required. Each component was coded as a binary variable (yes = 1, no = 0) and summed to generate a BPCR score ranging from 0 to 5, with higher scores indicating greater preparedness.

To assess the robustness of the findings and address potential concerns related to outcome definition, a sensitivity analysis was conducted using a binary BPCR classification. Women were categorized as well prepared if they reported practicing two or more BPCR components and poorly prepared if they practiced fewer than two components, consistent with commonly used definitions in the literature. This sensitivity analysis addressed outcome specification rather than exposure timing. BPCR practices were assessed with reference to each woman’s most recent pregnancy. Several explanatory variables, including participation in model family training, women’s decision-making autonomy, and selected community-level characteristics, were measured at the time of the survey and may not perfectly correspond to conditions during the index pregnancy. These variables were therefore interpreted as reflecting relatively stable individual and contextual characteristics in this setting, rather than precise time-specific exposures. This sensitivity analysis addressed outcome specification rather than exposure timing.

Independent variables were grouped into individual- and community-level factors. Individual-level variables included socio-demographic and economic characteristics (maternal and spouse age, education, occupation, household wealth index, and exposure to mass media) and obstetric history (gravidity, parity, previous stillbirth, and maternal age at marriage). Household socio-economic status was assessed using a wealth index constructed through principal component analysis (PCA), following standard demographic and health survey methodologies. The index was based on 43 household asset and living-condition indicators, including ownership of durable goods (e.g., radio, television, mobile phone), housing characteristics (roof, wall, and floor materials), access to water and sanitation facilities, sources of cooking fuel, land ownership, and livestock holdings. Variables were first standardized, and components with eigenvalues greater than one were retained. The first principal component, which explained the largest proportion of variance, was used to generate a composite wealth score for each household. Households were then ranked and categorized into five wealth quintiles (lowest, second, middle, fourth, and highest). Detailed measurement definitions are provided in Table S1 of Supplementary File S2.

**Community-level variables**: Community-level factors were derived by aggregating individual responses within each cluster (*kebele*) to capture the broader contextual environment influencing BPCR:

**Women’s literacy:** Calculated as the proportion of women in the cluster with at least primary education. Clusters were classified as “high literacy” if more than 50% of women had primary or higher education, and “low literacy” otherwise.

**Poverty:** Defined as the proportion of households in the poorest and poorer wealth quintiles within each cluster. Clusters with more than 50% of households in these lower two quintiles were categorized as “high poverty,” while the remainder were considered “low poverty.”

**Mass media exposure:** Measured as the proportion of women in the cluster who accessed at least one form of mass media (radio, television, or newspaper). Clusters were classified as “high exposure” if over 50% of women used any mass media, and “low exposure” otherwise.

**Distance to the nearest health facility:** Individual women were coded as “close” if they could reach the nearest health facility within 30 min on foot, and “far” otherwise. At the cluster level, communities were classified as “not a big problem” if more than 50% of women were close to a health facility, and “a big problem” if not.

### 2.8. Data Collection Procedures

Data were collected using a structured, pretested questionnaire adapted from previous studies (Supplementary File S3). The tool was developed in English, translated into Sidaamu Afoo (local language), and back-translated to ensure accuracy. Discrepancies were resolved through review by the principal investigator (PI) and a bilingual expert. Data collectors and supervisors received two days of training covering study objectives, data collection procedures, and ethical considerations. Pretesting on 5% of the sample in a neighboring district informed minor revisions. Data were collected face-to-face at participants’ homes using the Open Data Kit mobile application by trained health professionals fluent in Sidaamu Afoo (local language). For participants with low literacy, trained data collectors conducted face-to-face interviews in the local language, using simple explanations and probing questions to ensure accurate understanding and responses. Daily checks ensured completeness and consistency, and data were archived on the KoboToolbox server before being exported to Stata version 18 for cleaning, coding, and analysis.

### 2.9. Statistical Analysis

Categorical variables were summarized as frequencies and percentages, and continuous variables as means ± standard deviation after testing for normality. The household wealth index was constructed using PCA based on 43 indicators of household assets, housing, land size, livestock, fuel, and water and sanitation facilities (Table S2, Supplementary File S2).

BPCR scores were count data; preliminary Poisson regression indicated over-dispersion (mean = 1.37, variance = 1.58), violating the equidispersion assumption. Consequently, a multilevel mixed-effects negative binomial regression model was fitted to account for both within- and between-cluster variability. A series of nested models were fitted to identify the most appropriate model for the data. These included an empty (null) model without predictors, a model including individual-level variables only, a model including community-level variables only, and a full model incorporating both individual- and community-level predictors. Model comparison and selection were guided by multiple criteria, including the Akaike Information Criterion (AIC), Bayesian Information Criterion (BIC), log-likelihood values, and likelihood ratio tests. Models with lower AIC and BIC values and higher log-likelihoods were considered to demonstrate better fit. Measures of cluster-level variation, including the intraclass correlation coefficient (ICC) and median incidence rate ratio (MIRR), were also examined to assess the contribution of community-level effects. The final model was selected based on overall goodness-of-fit, parsimony, and interpretability. Potential confounding variables, including maternal age, parity, previous obstetric history, household wealth, and exposure to mass media, were considered in the analysis. Variables with *p* < 0.25 in bivariable analysis or with established clinical relevance were included in the multivariable multilevel mixed-effects negative binomial regression model. Adjusted incidence rate ratios (AIRRs) were estimated, allowing each statistically significant determinant such as occupation, planned pregnancy, model family training, autonomy, urban residence, and community literacy to reflect its independent effect on BPCR practice while controlling for potential confounders. This approach minimized bias and ensured robust estimation of the associations between determinants and maternal preparedness. Effect modification was assessed using interaction terms, and multicollinearity was checked via variance inflation factors (VIF < 5). Effect estimates are reported as AIRRs with corresponding 95% confidence intervals (CIs), reflecting the relative difference in the expected number of BPCR components associated with each determinant. Statistical significance was determined when the 95% CI did not include 1. As a sensitivity analysis, BPCR was additionally dichotomized using a conventional threshold (adequately prepared: ≥2 components; poorly prepared: <2 components), and a multilevel multivariable logistic regression model was fitted. Results from the sensitivity analysis are presented as adjusted odds ratios (AORs) with 95% CIs to assess the robustness of findings across alternative outcome specifications. Because precise information on the time elapsed since the most recent birth was not collected, time-restricted analyses or sensitivity analyses based on recency of delivery could not be conducted.

### 2.10. Ethical Considerations

Ethical approval was obtained from the Institutional Review Board of the College of Medicine and Health Sciences, Hawassa University. Permission was secured from the Sidama Regional Health Bureau, Hawela Lida District Health Office, and local kebele administrations. Written informed consent was obtained from all participants after providing detailed information on the study’s objectives, procedures, potential risks, and benefits. No personal identifiers were collected, and data were stored on password-protected servers accessible only to the research team, ensuring confidentiality.

## 3. Results

### 3.1. Study Population

A total of 3526 mothers participated in the study (Table 1). Fourteen eligible women declined participation or could not be reached after repeated visits. Available information from household listings indicated that non-respondents did not differ markedly from respondents in terms of age group, place of residence, or marital status. The mean maternal age was 26.0 ± 4.6 years, ranging from 15 to 49 years. The majority were of Sidama ethnicity (94%) and identified as Protestant Christians (85%), with nearly all currently married (99%). While 70% of participants had completed at least primary education, only 13% attained secondary education or higher. Most women were housewives (86%), whereas the predominant occupation among husbands was farming (55%). Household size was typically small, with four in five families comprising five or fewer members. Approximately 40% of respondents reported regular exposure to mass media. The asset-based wealth index was relatively evenly distributed across quintiles.

### 3.2. Reproductive and Obstetric Characteristics

The mean age at first marriage and first pregnancy were 21.2 ± 3.1 and 22.4 ± 3.2 years, respectively (Table 2). Most women (69%) had experienced two to four pregnancies, and 17.8% reported at least one prior abortion. Previous stillbirth was rare (1.6%). Although 83.4% of pregnancies were planned, 9.5% of mothers reported experiencing at least one obstetric danger sign during pregnancy.

### 3.3. Distribution of Birth Preparedness and Complication Readiness Scores

**Figure 1:**
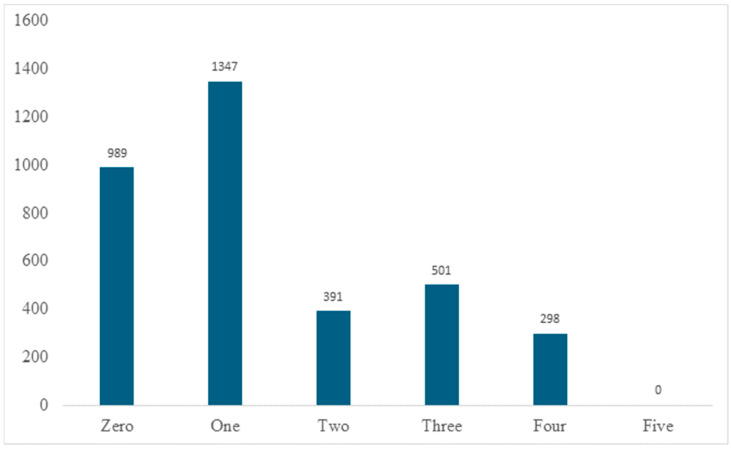
Distribution of birth preparedness and complication readiness scores among women of reproductive age in Hawela Lida District, Sidama Region, Ethiopia, 2025 (*n* = 3526).

The BPCR scores among the study population showed considerable variation (Figure 1). Of the 3526 women, 28.0% (*n* = 989) had a score of 0, indicating no birth preparedness activities, while 38.2% (*n* = 1347) reported a score of 1. Scores of 2 and 3 were observed in 11.1% (*n* = 391) and 14.2% (*n* = 501) of women, respectively. Only 8.5% (*n* = 298) achieved a score of 4, and none reached the maximum score of 5.

**Figure 2:**
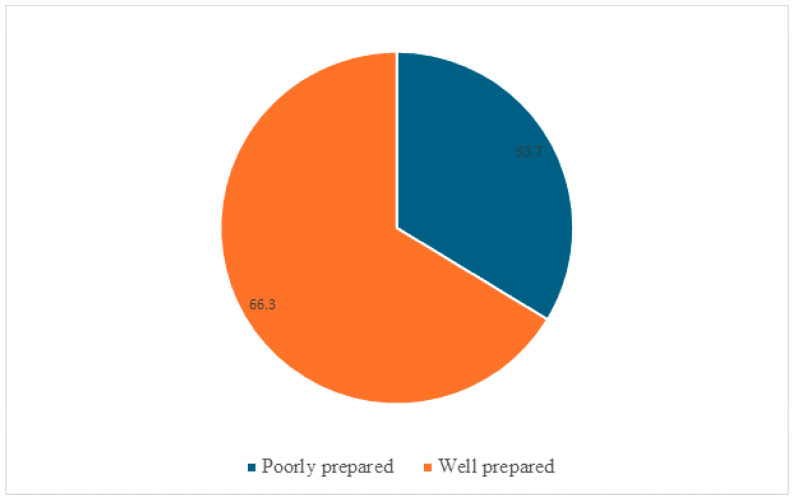
Proportion of birth preparedness and complication readiness status among women of reproductive age in the Hawela Lida district, Sidama Region, Ethiopia, 2025 (*n* = 3526).

Overall, the distribution indicates that most women engaged in limited BPCR practices, highlighting substantial gaps in maternal preparedness and potential barriers to accessing timely obstetric care. Based on the conventional categorization, 1190 women (33.7%) were classified as well prepared (BPCR ≥ 2), while 2336 women (66.3%) were considered poorly prepared (BPCR < 2). These findings indicate that only about one-third of women were adequately prepared for birth and potential complications, highlighting substantial gaps in maternal health preparedness in the study area (Figure 2).

### 3.4. Determinants of Birth Preparedness and Complication Readiness

Several individual- and community-level factors were significantly associated with the expected number of BPCR components (Table 3). At the individual level, women employed in government positions had an estimated 3.11 times higher expected BPCR score compared with farmers (AIRR = 3.11; 95% CI: 1.89–5.77), likely reflecting greater financial stability and improved access to health information. Women whose most recent pregnancy was planned had a 1.66-fold higher expected BPCR score than those with unplanned pregnancies (AIRR = 1.66; 95% CI: 1.15–3.22), indicating that pregnancy planning facilitates resource allocation and timely antenatal care. Participation in model family training more than doubled the expected BPCR score (AIRR = 2.53; 95% CI: 1.76–4.99), suggesting that structured training enhances knowledge of danger signs, emergency preparedness, and maternal care. Autonomous women exhibited a 2.34-fold higher expected BPCR score than non-autonomous counterparts (AIRR = 2.34; 95% CI: 1.97–5.93), highlighting the importance of decision-making power in arranging care, transport, and financial resources.

At the community level, urban residence was associated with a 2.78-fold higher expected BPCR score (AIRR = 2.78; 95% CI: 1.81–4.77), reflecting better infrastructure, healthcare access, and exposure to health promotion. Similarly, women living in communities with high literacy levels had nearly five times higher expected BPCR scores compared with low-literacy communities (AIRR = 4.92; 95% CI: 2.32–8.48), indicating the role of social norms and information dissemination in facilitating maternal preparedness. Detailed multivariable regression results, including coefficient estimates, are provided in Supplementary File S4.

### 3.5. Sensitivity Analysis Result

In the sensitivity analysis using a binary BPCR outcome (well prepared ≥ 2 components vs. poorly prepared < 2), the multilevel logistic regression yielded results consistent with the primary negative binomial model. Government employment, planned pregnancy, women’s decision-making autonomy, participation in model family training, urban residence, and higher community-level women’s literacy remained independently associated with higher odds of being well prepared for birth and potential complications. The consistency in direction and statistical significance across both modeling approaches supports the robustness of the study findings (Table 4).

### 3.6. Random Effect Model of Birth Preparedness and Complication Readiness

The multilevel mixed-effects negative binomial regression model provided a superior fit compared with the ordinary negative binomial model (*p* < 0.001). The intraclass correlation coefficient (ICC) indicated that 19.4% of the variability in BPCR practice was attributable to *kebele*-level clustering. The MIRR demonstrated that residual heterogeneity between residential areas corresponded to a 2.31-fold difference in the likelihood of BPCR when randomly selecting two individuals from different *kebeles*. Even after adjusting for all individual- and community-level predictors, significant heterogeneity in BPCR practice across *kebeles* persisted. Additionally, the effect of model family training on BPCR practice varied significantly across *kebeles* (variance = 0.64; 95% CI: 0.15–3.55).

### 3.7. Model Selection Criteria

Model fit statistics indicated that the empty (null) model, which included no predictors, provided the poorest fit to the data (AIC = 3115.77, BIC = 3138.66, log-likelihood = −1559.88). Successive models incorporating individual-level and community-level predictors showed progressive improvement, reflected in decreasing AIC and BIC values and increasing log-likelihoods. The final multilevel mixed-effects negative binomial model, which included both individual- and community-level factors, demonstrated the best overall fit (AIC = 2915.15, BIC = 3003.33, log-likelihood = −1402.44). Additionally, likelihood ratio tests confirmed that this model was significantly superior to simpler alternatives. Based on these criteria, along with considerations of parsimony and interpretability, the final model was selected as the most appropriate representation of the data.

## 4. Discussion

This study investigated the individual- and community-level determinants of birth preparedness and complication readiness among women of reproductive age in the Hawela Lida district of Sidama Region, Ethiopia, using a multilevel mixed-effect negative binomial regression model. The findings highlight important determinants of BPCR practices, revealing that both individual-level factors, such as occupation, pregnancy planning, autonomy, and participation in model family training, and community-level characteristics, including residence and women’s literacy, significantly shape maternal readiness for childbirth and obstetric emergencies. These results not only align with previous evidence but also expand current understanding of how multilevel dynamics influence maternal health behaviors in Ethiopia and similar contexts.

### 4.1. Individual-Level Determinants of BPCR

Women employed in government positions had over three times higher expected BPCR scores compared with farmers. This association is consistent with studies conducted in northern Ethiopia [26], northwest Ethiopia [27], and Ghana [28], which reported higher BPCR practices among formally employed women. Employment likely enhances women’s financial autonomy, access to information, and decision-making power, all of which facilitate proactive planning for childbirth. In contrast, women engaged in subsistence farming often face economic insecurities, limited cash flow, and restricted access to health information, which may reduce their ability to save money, arrange transport, or identify skilled birth attendants. The discrepancy between government employees and farmers in BPCR engagement may also reflect structural inequities in occupational status, as government-employed women are more integrated into systems that provide exposure to health promotion messages and institutional maternal health initiatives.

Pregnancy intention emerged as an important determinant, with women who planned their most recent pregnancy exhibiting higher expected BPCR scores compared with those whose pregnancies were unplanned. This finding corroborates results from the 2019 Ethiopian Mini Demographic and Health Survey [29] and studies from Tanzania [30] and Nepal [31]. Planned pregnancies allow families to allocate resources, mobilize social support, and engage more fully with antenatal care (ANC) services. In contrast, unplanned pregnancies may be associated with delayed ANC initiation, reduced maternal motivation to prepare, and psychosocial stress, which collectively undermine readiness for birth and complications. However, some studies in rural Uganda reported weaker associations between pregnancy planning and BPCR [32], possibly due to differences in cultural norms surrounding fertility, where pregnancies are socially welcomed regardless of intention, thereby minimizing behavioral differences between planned and unplanned conceptions.

Participation in model family training was associated with more than a twofold increase in the expected BPCR score, indicating that trained women engaged in a greater number of birth preparedness activities compared with those who had not received training. This association is consistent with findings from previous studies conducted in Sidama and Oromia [33,34], which found that households that completed health extension packages demonstrated improved maternal and child health outcomes. Model family training equips households with practical knowledge on maternal nutrition, antenatal and postnatal care, and emergency preparedness. Its effectiveness can be attributed to both knowledge transfer and the role of trained families as role models within their communities, creating positive peer influence. Nevertheless, studies from northern Ethiopia noted variability in the effectiveness of such programs depending on program fidelity and community engagement [35]. Thus, while the present findings confirm the value of community-based training models, they also suggest the need for continuous quality assurance and contextual adaptation.

Women who reported autonomy in household decision-making exhibited more than a twofold higher expected BPCR score compared with non-autonomous women, highlighting the critical role of empowerment in facilitating engagement with birth preparedness activities. These findings echo findings from studies in Ethiopia [36], Nigeria [37], and India [38], which consistently demonstrate that women’s empowerment is central to maternal health-seeking behaviors. Autonomy enhances women’s control over financial resources, freedom of movement, and ability to seek skilled care, thereby directly improving preparedness for childbirth and emergencies. In patriarchal contexts, restricted autonomy often delays critical decisions, such as arranging transport or seeking care when complications arise, which has been described as a “second delay” in the three-delay model [39]. Importantly, interventions that promote women’s decision-making power, including community dialogues and male-involvement strategies, have been shown to increase BPCR in diverse settings [40].

### 4.2. Community-Level Determinants of BPCR

At the community level, residing in an urban area was associated with nearly a threefold higher expected BPCR score compared with rural residence, underscoring the influence of infrastructure, healthcare accessibility, and exposure to health information on maternal preparedness. This result aligns with multiple Ethiopian studies, including those conducted in the Southern Nations, Nationalities, and Peoples’ Region [41], Tigray [42], and Addis Ababa [43], where urban women consistently demonstrated higher BPCR. Similar findings have been reported in Nigeria [44], Bangladesh [45], and Kenya [46]. The urban–rural divide likely reflects disparities in health infrastructure, road access, transportation availability, and exposure to health information through mass media. Rural women often face physical and financial barriers that constrain their ability to prepare, even when they possess adequate knowledge. This persistent inequity highlights the importance of geographically targeted interventions, such as maternity waiting homes, community transport schemes, and rural-focused health education campaigns.

Community-level literacy emerged as a strong predictor, with women living in highly literate communities having nearly fivefold higher expected BPCR scores compared with those in communities with lower literacy, highlighting the role of social and educational environments in shaping maternal preparedness. This finding resonates with evidence from Ethiopia [47], Malawi [48], and Nepal [49], where collective literacy environments have been shown to shape individual health behaviors. High community literacy fosters shared norms of health-seeking, increases the likelihood of peer discussions around maternal care, and facilitates comprehension of health messages delivered through written and broadcast media. Even women with limited personal education may benefit from living in literate communities through indirect knowledge transfer and collective action. Conversely, in low-literacy settings, limited exposure to information constrains not only individual readiness but also community mobilization for maternal health. The large effect size observed in this study underscores the importance of addressing literacy as a structural determinant of maternal health, beyond individual-level education.

### 4.3. Comparison with Other Studies in Ethiopia and Beyond

Overall, the determinants identified in this study—occupation, pregnancy intention, participation in model family training, autonomy, residence, and community literacy—are broadly consistent with findings from other Ethiopian and international studies. However, variations in magnitude and significance highlight the complex interplay of cultural, socio-economic, and health-system contexts. For instance, while autonomy has been universally associated with BPCR, its effect size is larger in patriarchal societies with limited gender equity [36,37], whereas in relatively egalitarian contexts, the association may be weaker [38]. Similarly, the strong influence of community literacy observed in this study may reflect the district’s reliance on interpersonal communication for health promotion, contrasting with urban centers where individual education plays a more dominant role.

Some discrepancies between this study and others may also arise from methodological differences. Whereas many previous Ethiopian studies employed simple logistic regression, our use of multilevel mixed-effect negative binomial modeling accounts for clustering and over-dispersion, producing more precise estimates. Such methodological improvements may partly explain why community-level factors such as literacy emerged with greater magnitude in our findings compared to earlier research [41,42].

### 4.4. Discussion of Contextual Variables and Ecological Fallacy

In this study, community-level variables such as women’s literacy, poverty, mass-media exposure, and distance to health facilities were included to capture the broader social and environmental factors shaping BPCR. These aggregated measures provide valuable insight into the contextual influences that go beyond individual characteristics. However, it is important to recognize the risk of ecological fallacy: the observed associations at the community level do not necessarily imply the same relationships hold at the individual level. For example, residing in a highly literate community may create supportive norms and access to information that facilitate BPCR, yet this does not automatically ensure that every woman in that community is well-prepared. By explicitly acknowledging this limitation, our study highlights the importance of interpreting community-level effects as contextual influences rather than direct individual determinants [24,50]. This perspective underscores the need for multilevel interventions that address both personal empowerment and broader community conditions to improve maternal preparedness and reduce inequities in maternal health outcomes.

### 4.5. Implications for Policy and Practice

The findings of this study have important implications for maternal health policy and practice in Ethiopia. Firstly, enhancing women’s economic opportunities and employment, particularly beyond subsistence farming, can strengthen birth preparedness by increasing financial autonomy, access to health information, and the ability to make timely decisions. Secondly, promoting pregnancy planning through robust family planning programs can reduce the incidence of unplanned pregnancies, supporting better preparation for childbirth and potential complications. Thirdly, scaling up model family training across communities is essential, but must be paired with quality monitoring and supervision to ensure consistent knowledge transfer and practical application. Fourthly, interventions that foster women’s decision-making autonomy and challenge entrenched patriarchal norms are critical for improving maternal health behaviors. Finally, addressing structural barriers such as limited rural health infrastructure, inadequate transportation, and low community literacy through targeted programs can create supportive environments that enhance BPCR practices. Collectively, these strategies provide actionable guidance for policymakers and program planners seeking to reduce maternal morbidity and mortality and strengthen the maternal health system in Ethiopia.

### 4.6. Strengths and Limitations

The strengths of this study include its large sample size, rigorous multistage sampling, and use of advanced multilevel modeling to disentangle individual and community effects. Nevertheless, certain limitations should be acknowledged. The cross-sectional design limits causal inference, and reliance on self-reported measures may introduce recall or social desirability bias. In addition, several explanatory variables particularly women’s decision-making autonomy, participation in model family training, and selected community-level characteristics were measured at the time of the survey and may not perfectly reflect conditions during the index pregnancy, introducing potential temporal mismatch and non-differential exposure misclassification. Furthermore, some unmeasured community-level variables, such as cultural beliefs and health-system quality, may partly explain the residual heterogeneity observed across *kebeles.* A further limitation is the lack of precise data on time since last birth, which precluded presenting a distribution or conducting time-restricted sensitivity analyses. Nevertheless, by anchoring BPCR measurement to the most recent pregnancy, we sought to minimize recall bias. Future studies may benefit from collecting detailed birth timing and longitudinal exposure data to allow for more refined temporal analyses. Despite these limitations, the study provides robust evidence with clear implications for maternal health programming in Ethiopia and similar settings.

## 5. Conclusions

This study highlights that both individual- and community-level factors shape BPCR in the Northern Zone of Sidama Region. Key individual determinants associated with BPCR include occupational status, pregnancy intention, women’s autonomy, and participation in model family training, while urban residence and high community literacy serve as important contextual drivers. These findings underscore the need for integrated interventions that enhance women’s empowerment, expand model family training, and address structural barriers such as rural access and low literacy. Policymakers and maternal health programs can leverage these insights to design targeted strategies that reduce maternal morbidity and mortality. Future research should evaluate the effectiveness of these interventions and explore their long-term impact on maternal and neonatal outcomes to further inform evidence-based health policies in Ethiopia.

## Figures and Tables

**Table 1:** Socio-demographic and economic characteristics of mothers, Hawela Lida district, southern Ethiopia, 2025 (*n* = 3526).

Variable	Category	*n*	%
Maternal age, y	15–49 (mean ± SD)	26.0 ± 4.6	—
Ethnicity	Sidama	3328	94.4
	Amhara	125	3.5
	Wolayita	38	1.1
	Gurage	35	1.0
Religion	Protestant Christian	2991	84.8
	Orthodox Christian	236	6.7
	Catholic	172	4.9
	Muslim	99	2.8
	Other	28	0.8
Maternal education	Cannot read/write	375	10.6
	Read/write only	75	2.1
	Primary (1–8)	2485	70.5
	Secondary (9–12)	363	10.3
	College diploma	119	3.4
	University ≥ degree	109	3.1
Maternal occupation	Housewife	3040	86.2
	Government employee	278	7.9
	Merchant	27	0.8
Marital status	Married	3499	99.2
	Div./sep./widowed	27	0.8
Husband occupation	Farmer	1935	55.3
	Merchant	1028	29.4
	Government employee	311	8.9
	Daily labourer	172	4.9
	Private employee	21	0.6
	Other	32	0.9
Husband education	Cannot read/write	120	3.4
	Read/write only	473	13.5
	Primary (1–8)	1932	55.2
	Secondary (9–12)	707	20.2
	College diploma	159	4.5
	University ≥ degree	108	3.1
Household size	1–5 members	2777	78.8
	>5 members	749	21.2
Mass-media exposure	Yes	1399	39.7
	No	2127	60.3
Wealth quintile	Lowest	717	20.3
	Second	730	20.7
	Middle	682	19.3
	Fourth	669	19.0
	Highest	728	20.6

Note: Read/write only refers to participants who can read and write but have not received formal education.

**Table 2:** Reproductive and obstetrics characteristics of mothers, Hawela Lida district, southern Ethiopia, 2025 (*n* = 3526).

Variable	Category	*n*	%
Age at first marriage, y	21.2 ± 3.1	—	—
Age at first pregnancy, y	22.4 ± 3.2	—	—
Number of gravidities	1	911	25.8
	2–4	2439	69.2
	≥5	176	5.0
Previous abortion	Yes	628	17.8
	No	2898	82.2
Previous stillbirth	Yes	55	1.6
	No	3471	98.4
Pregnancy planned	Yes	2942	83.4
	No	584	16.6
Obstetric danger signs during pregnancy	Yes	334	9.5

**Table 3:** Determinants of birth preparedness and complication readiness practice among women of reproductive age in the Hawela Lida district of Sidama region, Ethiopia, 2025 (*n* = 3526).

Variables	CIRR (95% CI)	AIRR (95% CI)
**Individual-level determinants**		
Women’s education status		
Cannot read and write	1	1
Can read and write only (no formal education)	0.96 (0.72, 1.26)	0.75 (0.71, 1.18)
Have a formal education	1.25 (1.18, 1.79)	1.07 (0.77, 1.40)
Women’s occupational status		
Housewife	1	1
Farmer	0.96 (0.72, 2.11)	0.85 (0.65, 2.30)
Government employee	4.21 (1.54, 7.47)	3.11 (1.89, 5.77) **
Merchant	1.33 (1.01, 2.33)	1.12 (0.70, 1.38)
Age at first pregnancy ^†^	1.42 (1.11, 1.74)	0.99 (0.66, 1.22)
Previous history of abortion		
No	1	1
Yes	1.55 (0.99, 3.10)	1.16 (0.88, 2.45)
Previous history of stillbirth		
No	1	1
Yes	2.12 (1.89, 3.10)	0.99 (0.67, 2.16)
Previous history of neonatal death		
No	1	1
Yes	2.21 (0.92, 3.55)	0.97 (0.62, 2.05)
Current pregnancy status		
Unplanned	1	1
Planned	2.59 (2.02, 3.18)	1.66 (1.15, 3.22) **
Faced a health problem during pregnancy		
No	1	1
Yes	2.41 (2.19, 2.98)	1.19 (0.99, 1.55)
Faced a health problem during childbirth		
No	1	1
Yes	2.32 (1.52, 2.99)	1.94 (0.99, 2.95)
Women’s decision-making power		
Non-autonomous	1	1
Autonomous	4.18 (2.03, 7.36)	2.34 (1.97, 5.93) **
Received model family training		
No	1	1
Yes	3.62 (2.18, 6.23)	2.53 (1.76, 4.99) **
**Cluster-level determinants**		
Place of residence		
Rural	1	1
Urban	5.34 (2.92, 9.92)	2.78 (1.81, 4.77) *
Cluster-level distance to nearest health facility		
Big problem	1	1
Not a big problem	1.98 (1.22, 3.11)	1.38 (0.99, 1.92)
Cluster-level women’s literacy		
Low	1	1
High	5.61 (3.41, 9.65)	4.92 (2.32, 8.48) *
Cluster-level mass media use		
Low	1	1
High	1.55 (0.93, 2.76)	1.05 (0.84, 1.53)

*: significant association (*p* < 0.05); **: highly significant association (*p* < 0.01); ^†^: continuous variable; CI: confidence interval; CIRR: crude incidence rate ratio; AIRR: adjusted incidence rate ratio; 1: reference group.

**Table 4:** Multilevel multivariable logistic regression of birth preparedness and complication readiness among women of reproductive age in Hawela Lida District, Sidama Region, Ethiopia, 2025 (*n* = 3526).

Variables	Crude Odds Ratio (COR) (95% CI)	Adjusted Odds Ratio (AOR) (95% CI)
Individual-level determinants		
Women’s education status		
Cannot read and write	1	1
Can read and write only	0.91 (0.73–1.14)	0.83 (0.65–1.07)
Formal education	1.29 (1.01–1.64)	1.12 (0.86–1.45)
Women’s occupational status		
Housewife	1	1
Farmer	0.89 (0.64–1.23)	0.81 (0.58–1.14)
Government employee	4.63 (2.38–9.01)	3.45 (1.92–6.21) **
Merchant	1.41 (0.99–2.01)	1.18 (0.81–1.71)
Age at first pregnancy (years) ^†^	1.11 (0.94–1.32)	1.02 (0.86–1.21)
Previous history of abortion		
No	1	1
Yes	1.28 (0.96–1.72)	1.14 (0.84–1.55)
Previous history of stillbirth		
No	1	1
Yes	1.41 (0.98–2.03)	1.01 (0.70–1.47)
Previous neonatal death		
No	1	1
Yes	1.36 (0.88–2.11)	0.97 (0.63–1.50)
Current pregnancy status		
Unplanned	1	1
Planned	2.41 (1.92–3.02)	1.74 (1.23–2.46) **
Faced health problem during pregnancy		
No	1	1
Yes	1.62 (1.21–2.16)	1.18 (0.94–1.49)
Faced health problem during childbirth		
No	1	1
Yes	1.58 (1.09–2.29)	1.32 (0.93–1.89)
Women’s decision-making power		
Non-autonomous	1	1
Autonomous	3.96 (2.51–6.24)	2.61 (1.83–3.72) **
Received model family training		
No	1	1
Yes	3.21 (2.17–4.76)	2.68 (1.89–3.79) **
Community-level determinants		
Place of residence		
Rural	1	1
Urban	4.89 (3.01–7.94)	2.94 (1.98–4.36) *
Distance to nearest health facility		
Big problem	1	1
Not a big problem	1.66 (1.17–2.35)	1.29 (0.97–1.72)
Community women’s literacy		
Low	1	1
High	5.02 (3.32–7.58)	4.11 (2.46–6.87) *
Community mass media use		
Low	1	1
High	1.41 (0.98–2.02)	1.09 (0.82–1.46)

*: significant association (*p* < 0.05); **: highly significant association (*p* < 0.01); ^†^: continuous variable; CI: confidence interval; COR: crude odds ratio; AOR: adjusted odds ratio; 1: reference group.

## Data Availability

Data are available at supplementary file S5.

## Supplementary Materials

The following supporting information can be downloaded at: https://www.mdpi.com/article/10.3390/epidemiologia7010013/s1 Supplementary File S1: STROBE reporting guidelines for cross-sectional study (DOCX); Supplementary File S2: Detailed information from methods sections (DOCX); Supplementary File S3: English version study questionnaire (DOCX); Supplementary File S4: The full multivariable regression output, including coefficient estimates, Supplementary File S5: De-identified dataset, which was authorized to be available by the institutional ethics committee and supported by informed consent from all study participants (dta).

## Abbreviations

The following abbreviations are used in this manuscript:

AIC	Akaike information criteria
AIRRs	Adjusted incidence rate ratios
BIC	BIC: Bayesian Information Criterion
BPCR	Birth preparedness and complication readiness
CI	confidence interval
CIRRs	Crude incidence rate ratios
HEW	Health Extension Workers
ICC	Intra-class correlation coefficient
LMICs	low- and middle-income countries
SD	Standard deviation
PCA	principal component analysis
WRA	women of reproductive age
VIF	Variance inflation factor.
