# Supplementary material for: Individual- and Community-Level Predictors of Birth Preparedness and Complication Readiness: Multilevel Evidence from Southern Ethiopia
Source: Epidemiologia (Basel). 2026 Jan 14;7(1):13. doi: 10.3390/epidemiologia7010013 (PMC12821691; doi:10.3390/epidemiologia7010013)
Supplement: Supplementary file 1 [file epidemiologia-07-00013-s001.zip › Supplementary File S2.pdf]

S1 File Table 1: Description of study variables

Study variables	Description
BPCR practice	was measured using 5 components of the question as to whether or not the woman planned for her most recent pregnancy such as identified a closer proper HF for childbirth, founded and communicated an SBA, saved money, a material resource for childbirth, and other associated costs, prepared or arranged transportation to a proper HF in case of childbirth and obstetric emergency and identified and fixed the compatible blood group givers in case of blood requirements. Responses were recorded as count data.
Maternal knowledge regarding obstetric danger sign (ODS)	were measured using the 30 questions during three phases namely antepartum (9 questions), intra-partum (12 questions), and postpartum (9 questions). The correct answers were assigned a score of 1, while incorrect answers were assigned a score of 0. Lastly, the total knowledge scores range from 0 to 30. The study respondents who spontaneously mention at least 3 ODS during each phase were classified as having "good knowledge" and those who were able to spontaneously mention 2 or fewer ODS have "poor knowledge"
Spontaneous knowledge	is defined as the knowledge of study participants who can name or call an ODS without being read the name of that sign by data collectors. Merely true ODS spontaneously mentioned by study participants were recorded during the interview
Distance from the HF	is considered as close to HF if a woman is reported to travel less than 5 km or walking hours less than 30 minutes by foot to reach the nearest
Use of mass media	is generated by combining whether a study participant listens to the radio, watches television, and reads the newspaper and categorized as "yes" if the respondent is exposed to at least 1 of the 3 media and "no" otherwise

Family size	is defined as a total number of individuals existing in the household and is categorized as small when it is < 5, and large (≥ 5)
Formal education	is the education extending from primary to secondary and higher education and requires an organized and careful purpose that concretizes itself in an official curriculum, applied with a defined calendar and timetable [17].
Women's autonomy	a woman is considered autonomous if a woman can decide when and where to use MHS or on the health care spending by herself alone or with her husband together and a non-autonomous otherwise using a woman's self-report

Wealth index analysis method

The multiple response variables were categorized into binary responses (yes/no) and "I don't know" responses often coded as 999 to zero (Table 2). Similarly, the "I don't know" response and any missing value are often coded as 999 to zero for the continuous variables. The predictors that can differentiate between comparatively "poor" and "rich" households were selected using simple frequency analysis. Thus, our PCA didn't comprise any assets or variables that were possessed by less than 5% or more than 95% of the individuals in the sample. Finally, the component factors or wealth index scores were ranked into 5 classes such as lowest, second-lowest, middle, second highest, and highest. The PCA was carried out for the computation of the wealth index. All the basic assumptions of PCA were checked before ranking the components' factor scores into wealth quintiles. We removed the variables from PCA that didn't satisfy the assumptions such as the Kaiser-Meyer-Olkin (KMO) measure of sampling adequacy less than 0.5, commonalities less than 0.5, and variables that contain the complex structure (high loading correlation ≥0.4 on greater than one component).

S1 File Table 2: Some of variables and given values to facilitate the computation of wealth index

S.no	Variables	Given values
1	Main source of drinking water	Improved: Piped water, tube well or borehole, protected well, protected spring = 1<br>Unimproved: Unprotected well, Surface water (river and dam), Unprotected spring, Lake/pond/stream/canal = 0
2	Main source of water used for other purposes such as cooking and hand washing	Improved: Piped water, tube well or borehole, protected well, protected spring = 1<br>Unimproved: Unprotected well, Unprotected spring, Lake/pond/stream/canal, Surface water (River/dam) = 0
3	Where is that water source located?	In own dwelling or yard/plot = 1<br>Elsewhere = 0
4	Type of toilet facilities	Improved: comprise any non-shared toilet of the subsequent kinds: pour/flush toilets to septic tanks, piped sewer systems, and pit latrines; pit latrines with slabs; ventilated improved pit (VIP) latrines; and composting toilets = 1<br>Unimproved: Pit latrine without slab/open pit, bucket toilet and hanging toilet = 0
5	Where is this toilet facility located?	In own dwelling or yard/plot = 1<br>Elsewhere = 0
6	Type of fuel the household mainly use for cooking	Clean fuels include electricity, liquefied petroleum gas (LPG), natural gas, kerosene, and biogas = 1

		Solid fuels include coal, charcoal, wood, straw/shrub/grass, agricultural crops, and animal dung = 0
7	Where is the cooking usually done?	In the house and outdoors = 0<br>In a separate building = 1
8	Who is the owner of the house?	Me = 1<br>Rental, family, and relative = 0
9	Main material of the roof of the house	Natural roofing (no roof, mud, and sod) = 0<br>Rudimentary and finished roofing = 1
10	Main material of the floor of the house	Natural floor (Earth/sand, dung) = 0<br>Rudimentary and finished floor = 1
11	Main material of the wall of the house	Natural walls (no walls, cane/palm/trunks/bamboo/ree, dirt) = 0<br>Rudimentary and finished wall = 1
12	All other categorical variables were considered as yes and no form	Yes = 1 and no =0
13	All continuous variables were treated as continuous	
14	"I don't know" response often coded as 999 for categorical variables	999 = 0
15	"I don't know" response and any missing value often coded as 999 to zero	999 and missing value = 0
