# Supplementary material for: Individual- and Community-Level Predictors of Birth Preparedness and Complication Readiness: Multilevel Evidence from Southern Ethiopia
Source: Epidemiologia (Basel). 2026 Jan 14;7(1):13. doi: 10.3390/epidemiologia7010013 (PMC12821691; doi:10.3390/epidemiologia7010013)
Supplement: Supplementary file 1 [file epidemiologia-07-00013-s001.zip › Supplementary File S4.pdf]

Supplementary File 4. Full Multilevel Mixed-Effects Negative Binomial Regression Output for BPCR Practice Among Women of Reproductive Age in Hawela Lida District, Sidama Region, Ethiopia, 2025 (N = 3,526)

Variables	Coefficient (β)	Standard Error (SE)	Adjusted prevalence ratio (95% CI)	p-value
**Individual-level Determinants**				
Women's education status				
Cannot read and write	Ref	Ref	Ref	Ref
Can read and write only	-0.29	0.22	0.75 (0.71–1.18)	0.23
Formal education	0.07	0.20	1.07 (0.77–1.40)	0.65
Women's occupational status				
Housewife	Ref	Ref	Ref	Ref
Farmer	-0.16	0.26	0.85 (0.65–2.30)	0.48
Government employee	1.14	0.33	3.11 (1.89–5.77)	<0.001**
Merchant	0.11	0.21	1.12 (0.70–1.38)	0.41
Age at first pregnancy (years)	-0.01	0.11	0.99 (0.66–1.22)	0.97
Previous history of abortion				
No	Ref	Ref	Ref	Ref
Yes	0.15	0.22	1.16 (0.88–2.45)	0.39
Previous history of stillbirth				
No	Ref	Ref	Ref	Ref
Yes	-0.01	0.23	0.99 (0.67–2.16)	0.98
Previous history of neonatal death				
No	Ref	Ref	Ref	Ref
Yes	-0.03	0.22	0.97 (0.62–2.05)	0.92
Current pregnancy status				

Unplanned	Ref	Ref	Ref	Ref
Planned	0.51	0.22	1.66 (1.15–3.22)	0.01**
Faced health problem during pregnancy				
No	Ref	Ref	Ref	Ref
Yes	0.17	0.10	1.19 (0.99–1.55)	0.06
Faced health problem during childbirth				
No	Ref	Ref	Ref	Ref
Yes	0.66	0.34	1.94 (0.99–2.95)	0.05
Women's decision-making power				
Non-autonomous	Ref	Ref	Ref	Ref
Autonomous	0.85	0.26	2.34 (1.97–5.93)	<0.001**
Received model family training				
No	Ref	Ref	Ref	Ref
Yes	0.93	0.28	2.53 (1.76–4.99)	<0.001**
**Cluster-level Determinants**				
Place of residence				
Rural	Ref	Ref	Ref	Ref
Urban	1.02	0.32	2.78 (1.81–4.77)	0.002*
Cluster-level distance to nearest health facility				
Big problem	Ref	Ref	Ref	Ref
Not a big problem	0.32	0.17	1.38 (0.99–1.92)	0.06
Cluster-level women's literacy				
Low	Ref	Ref	Ref	Ref

High	1.59	0.45	4.92 (2.32–8.48)	<0.001*
Cluster-level mass media use				
Low	Ref	Ref	Ref	Ref
High	0.05	0.18	1.05 (0.84–1.53)	0.74

Notes:

- $p < 0.05$; **p** < 0.01
- APR: Adjusted Prevalence Ratio; SE: Standard Error; CI: Confidence Interval; ref: reference category.
- Model fitted using multilevel mixed-effects negative binomial regression, accounting for clustering at the kebele level.
